# Optical Coherence Tomography Angiography of Combined Central Retinal Artery and Vein Occlusion

**DOI:** 10.1155/2018/4342158

**Published:** 2018-02-12

**Authors:** Shuo-chieh Wu, Victor M. Villegas, Jaclyn L. Kovach

**Affiliations:** Department of Ophthalmology, Bascom Palmer Eye Institute, University of Miami Miller School of Medicine, Miami, FL, USA

## Abstract

Optical coherence tomography angiography (OCTA) is a new, noninvasive technology that enables detailed evaluation of flow in the retinal and choroidal vasculature. The authors believe this to be the first report to describe the optical coherence tomography angiography findings associated with combined central retinal artery occlusion (CRAO) and central retinal vein occlusion (CRVO).

## 1. Introduction

Combined central retinal artery occlusion (CRAO) and central retinal vein occlusion (CRVO) is a rare vasoocclusive entity that has been associated with multiple etiologies that can cause devastating vision loss [1–8]. In the population without age-related cardiovascular risk factors, the majority of the combined cases has been attributed to rheological causes, including thrombophilia, vessel wall inflammation, and mechanical compression [3].

Optical coherence tomography angiography (OCTA) is a new, fast, noninvasive imaging modality that allows detection of blood flow through the retinal and choroidal plexuses without intravenous dye injection [9]. The depth-resolved imaging technique affords insight regarding various retinal and choroidal diseases that is not available through other diagnostic modalities, such as fluorescein angiography (FA) [10]. OCTA is rapidly becoming an indispensable tool to describe a spectrum of pathologies, including macular degeneration, diabetic retinopathy, glaucoma, and choroidal neovascularization [10, 11]. Recently, it has been utilized as an adjunct tool to characterize retinal venous or arterial occlusion [10, 12, 13].

The authors believe this report to be the first to describe the optical coherence tomography angiography findings associated with combined central retinal artery occlusion and central retinal vein occlusion. The commercially available Cirrus 5000 with AngioPlex (Zeiss, Jena, Germany) was used, without any subsequent image modification or processing.

## 2. Case Report

A healthy 69-year-old female presented to the Emergency Department with sudden, painless, visual loss that started immediately following cataract surgery with retrobulbar anesthesia in the left eye (OS) nine days prior to presentation. The patient denied jaw claudication, temporal headache, scalp tenderness, or visual loss in the right eye (OD). Immediately following the event, the patient underwent a work-up which included a transthoracic echocardiogram (TTE), electrocardiogram (EKG), carotid ultrasound, erythrocyte sedimentation rate (ESR)/C-reactive protein (CRP), computed tomography (CT), and magnetic resonance imaging (MRI) of head. All tests were within normal limits.

A complete ophthalmologic exam was performed. Best corrected visual acuity was 20/40 OD and hand motion OS. Intraocular pressure measured by Tono-Pen XL (Reichert Technologies) was 18 mmHg OD and 19 mmHg OS. Full ductions were present without pain. Pupils were equally round with an afferent pupillary defect OS. Anterior segment examination in the right eye was significant for a nuclear sclerotic cataract and examination of the left eye revealed corneal edema, trace cell, +1 flare, and a well-centered intraocular lens.

**Figure 1:**
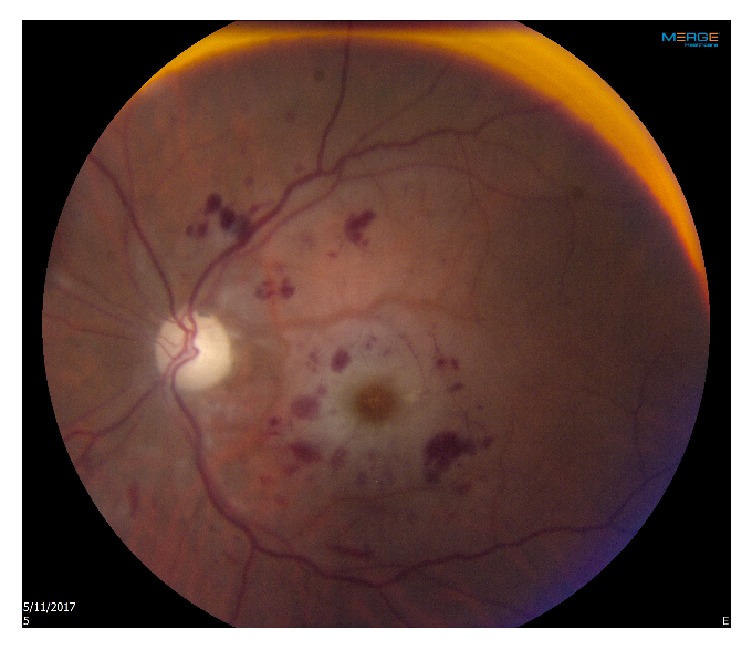
Fundus color photography of the left eye.

Fundus examination by indirect ophthalmoscopy was unremarkable OD. Funduscopic exam OS demonstrated mild disc edema, macular edema, whitening of the macula, subtle tortuosity of vessels, and flame-shaped hemorrhages and cotton wool spots in all quadrants (Figure 1).

**Figure 2:**
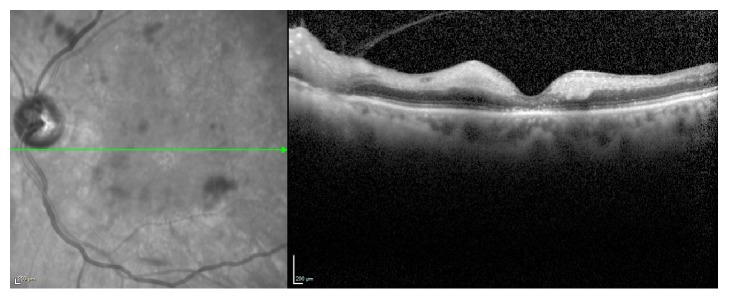
Spectral domain optical coherence tomography of the left eye.

Spectral domain optical coherence tomography (SD-OCT) was performed on OS and showed increased hyperreflectivity and edema of the inner retina with disruption of the ellipsoid zone (EZ) (Figure 2).

**Figure 3:**
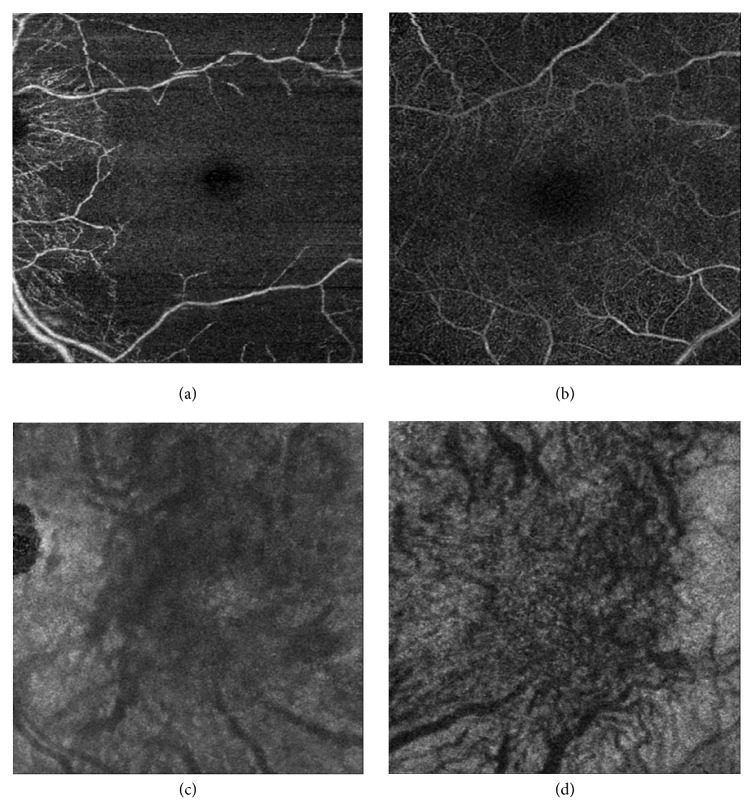
Optical coherence tomography angiography of the left eye. Vascular flow in the (a) superficial retinal capillary plexus, (b) deep retinal capillary plexus, (c) choriocapillaris plexus, and (d) choroidal plexus.

OCTA revealed an absence of flow in the foveal and perifoveal area in the superficial and deep retinal capillary plexuses (Figures 3(a) and 3(b)). In contrast, there is minimal alternation in choriocapillaris and choroidal vascular flow (Figures 3(c) and 3(d)).

## 3. Discussion

A combined CRAO and CRVO is a rare entity, and the etiology is incompletely understood. Although cardiovascular diseases, hypercoagulopathy, and inflammatory diseases are potential risk factors, our patient presented with a combined occlusion without any history of systemic diseases following cataract surgery with retrobulbar anesthesia [1–4]. Several studies have reported the occurrence of a combined CRAO and CRVO following retrobulbar injections, suggesting it can be a severe complication of periocular anesthesia [14–20].

The exact mechanism of combined CRAO and CRVO has not been elucidated, but there are multiple mechanisms proposed to explain the association with retrobulbar injection. Combined occlusion could result from optic nerve sheath hematoma secondary to needle penetration or direct injection into the optic nerve sheath [16, 21]. Another potential mechanism is the compromise of one circulation leading to the occlusion of the other. Brown et al. described two patients who initially presented with a CRVO and then developed a subsequent CRAO, suggesting that increased venous pressure could cross the capillary bed to impede the arterial flow and cause ischemia [18].

Combined CRAO and CRVO is an ophthalmological emergency that should be recognized as a serious postsurgical complication due to its poor outcome. Without timely intervention, combined occlusion can lead to rubeosis iridis, neovascular glaucoma, retinal necrosis, periphlebitis of the central vein, and eventually permanent vision loss [18, 22]. Various treatment modalities have been attempted to reverse the pathology with limited success, including triamcinolone, bevacizumab, and hyperbaric oxygen therapy. However, Vallée et al. demonstrated that timely intervention with fibrinolytics may restore retinal perfusion with visual improvement [2, 4, 22].

In the current report, OCTA showed that the vascular flow of the superficial and deep retinal plexuses were both interrupted OS (Figures 3(a) and 3(b)). In contrast, the choriocapillaris and choroidal vascular flow were minimally affected (Figures 3(c) and 3(d)). These results together suggest that the occlusion was limited to the retinal circulation without significant involvement of the choroidal circulation. Fluorescein angiography would have allowed us to assess the macular flow impairment. However, OCTA enables visualization of the flow disruption in the superficial and deep retinal capillary plexuses. With the depth of vascular disruption as a new metric for assessing disease severity, OCTA can provide more information regarding visual prognosis for this condition and other retinal vascular diseases.

This case demonstrates clinical features of combined CRAO and CRVO imaged with OCTA following retrobulbar anesthesia associated with cataract surgery. OCTA technology can facilitate diagnosis and extent of combined CRAO and CRVO as it enables discrimination between superficial and deep retinal vasculature. Additional advantages of OCTA compared to FA include faster image acquisition and no potential allergic systemic effects [9, 23, 24].

In conclusion, OCTA is a new, fast, noninvasive imaging technology that has enabled improved understanding of the pathophysiology of many retinal vascular diseases including combined CRAO and CRVO. To the best of our knowledge, this is the first reported case that describes the OCTA findings associated with combined CRAO and CRVO. Future studies with OCTA will hopefully illuminate additional features of combined CRAO and CRVO and provide a better understanding of this complex disease.
